# A phase II study of 5-fluorouracil/L-leucovorin/oxaliplatin (mFOLFOX6) in Japanese patients with metastatic or unresectable small bowel adenocarcinoma

**DOI:** 10.1007/s10147-017-1138-6

**Published:** 2017-05-23

**Authors:** Takahiro Horimatsu, Norisuke Nakayama, Toshikazu Moriwaki, Yoshinori Hirashima, Mikio Fujita, Masako Asayama, Ichiro Moriyama, Koji Nakashima, Eishi Baba, Hiroshi Kitamura, Takao Tamura, Ayumu Hosokawa, Kenichi Yoshimura, Manabu Muto

**Affiliations:** 1 Department of Therapeutic Oncology, Kyoto University Hospital, 54, Kawahara-cho, Shogoin, Sakyo-ku, Kyoto, 606-8507 Japan; 2 Department of Gastroenterology, Kanagawa Cancer Center, Kanazawa, Japan; 3 Division of Gastroenterology, University of Tsukuba, Tsukuba, Japan; 4 Department of Medical Oncology and Hematology, Faculty of Medicine, Oita University, Oita, Japan; 5 Department of Gastroenterology and Hepetology, Kobe City Medical Center General Hospital, Kobe, Japan; 6 Department of Gastroenterology, Saitama Cancer Center Hospital, Saitama, Japan; 7 Division of Clinical Study of Oncology, School of Medicine, Shimane University, Matsue, Japan; 8 First Department of Internal Medicine, Faculty of Medicine, University of Miyazaki, Miyazaki, Japan; 9 Department of Comprehensive Clinical Oncology, Faculty of Medical Sciences, Kyushu University, Fukuoka, Japan; 10 Department of Internal Medicine, Medical Oncology, School of Medicine, Kyorin University, Tokyo, Japan; 11 Department of Medical Oncology, Faculty of Medicine, Kinki University, Higashiosaka, Japan; 12 Department of Gastroenterology and Hematology, Faculty of Medicine, University of Toyama, Toyama, Japan; 13 Innovative Clinical Research Center (ICREK), Kanazawa University Hospital, Kanazawa, Japan

**Keywords:** Adenocarcinoma, Chemotherapy, mFOLFOX6, Immunochemical analysis, Oxaliplatin, Small bowel

## Abstract

**Background:**

Several studies have suggested that chemotherapy prolonged survival in patients with metastatic or recurrent small bowel adenocarcinoma (SBA); however, there is still no standard chemotherapy regimen. Here, we evaluated the efficacy and safety of a 5-fluorouracil (5-FU)/L-leucovorin (l-LV)/oxaliplatin (mFOLFOX6) protocol as a first-line therapy for patients with SBA.

**Patients and methods:**

This was a multicenter, single-arm, open-label phase II study. Eligibility criteria included histologically confirmed adenocarcinoma, age 20–80 years, and an Eastern Cooperative Oncology Group performance status (PS) of 0–2. The primary endpoint was 1-year progression-free survival (PFS). The secondary endpoints included overall response rate (ORR), overall survival (OS), overall PFS, and safety.

**Results:**

Between April 2010 and November 2012, 24 patients were enrolled from 12 institutions. The median age of the patients was 63 years (range 31–79) and there was a male/female ratio of 18/6. The number of PS 0/1 patients was 17/7 and locally advanced/metastatic disease was seen in 2/22 patients, respectively. The primary tumor site was the duodenum in 14 patients (58%) and jejunum in 10 patients (42%). The median follow-up time was 14.7 months (3.7–40.3). The 1-year PFS was 23.3%. The ORR was 9/20 (45%). The median PFS and OS times were 5.9 months (95% confidence interval [CI] 3.0–10.2) and 17.3 months (95% CI 11.7–19.0), respectively. Major grade 3/4 toxicities were neutropenia (38%), anemia/peripheral neuropathy (25%), and stenosis (17%). There were no treatment-related deaths.

**Conclusions:**

Although the primary endpoint was not met, mFOLFOX6 showed effective and good tolerance as a first-line treatment for SBA.

## Introduction

Adenocarcinoma of the small bowel (SBA) is a rare form of gastrointestinal cancer. SBA accounts for approximately one-third of all small intestinal malignancies, with the other major tumor types being neuroendocrine carcinomas, sarcomas, and lymphomas [1, 2]. The age-standardized incidence rates of SBA were reported to be 8.1 per million men and 5.5 per million women in the USA in 1995–2008 [3]. In Sweden, the age-standardized incidence of all malignant small bowel tumors (including adenocarcinomas, carcinoids, sarcomas, and lymphomas) increased from 14.2−19.7 per million people. In particular, the incidence of duodenal adenocarcinoma increased dramatically from 0.7−4.2 per million people during the period 1960–2009 [4].

As symptoms of SBA are usually nonspecific, diagnosis is difficult. Most affected patients present with advanced-stage disease and either lymph node involvement or distant metastatic disease [5]. Surgical management, with regional lymph node dissection is the only therapeutic modality with curative potential for localized SBA. For unresectable or recurrent tumors, chemotherapy regimens are generally used, as indicated for other gastrointestinal malignancies. Although several retrospective studies suggested that chemotherapy prolonged the survival of patients with unresectable or recurrent SBA [6–10], there are still no standard treatment protocols, and no randomized controlled trials have been carried out for these types of tumor [6–17].

Fluorouracil (5-FU) is the most commonly used agent for the treatment of unresectable or recurrent SBA, and various 5-FU-based regimens have been used [8–20]. In a retrospective analysis, Overman et al. reported that combination therapy with 5-FU and platinum compounds showed better results than other regimens [18]. Among the various types of combinations of 5-FU and platinum, oxaliplatin-containing regimens showed better efficacy in several studies. To date, two multicenter retrospective studies have been conducted. Zaanan et al. and Tsushima et al. reported median progression-free survival (PFS) times of 6.9 and 9.3 months, respectively, and median overall survival (OS) times of 17.8 and 22.2 months, respectively, with leucovorin + 5-FU + oxaliplatin (FOLFOX) therapy [19, 20]. In addition, two prospective studies have been reported. Overman et al. and Xiang et al. reported on CAPOX (capecitabine + oxaliplatin) therapy and FOLFOX4 therapy and found median times to treatment failure of 11.3 and 7.8 months, respectively, and median OS times of 20.4 and 15.2 months, respectively [21, 22]. Although combination therapy with 5-FU and oxaliplatin appears promising, to date the efficacy and safety of the mFOLFOX6 regimen (defined below) for SBA have not been demonstrated in any study. Therefore, we investigated the efficacy and safety of this regimen for Japanese patients with unresectable or recurrent SBA.

## Patients and methods

### Patients

All eligible patients were required to have histologically confirmed unresectable or recurrent SBA, excluding any ampullary carcinomas. Inclusion criteria were age 20–80 years; Eastern Cooperative Oncology Group (ECOG) performance status (PS) of 0–2; and adequate hematologic parameters (white blood cell count ≥3000 and ≤12,000/mm^3^, neutrophil count ≥1500 cells/mm^3^, platelet count ≥100,000 cells/mm^3^, and hemoglobin ≥8 g/dL), normal hepatic function (total bilirubin ≤2.0 g/dL, and transaminases ≤100 IU/L), and normal renal function (creatinine ≤1.5 mg/dL). Prior chemotherapy or radiotherapy was not allowed, but prior use of adjuvant chemotherapy at least 6 months before evidence of recurrence was permitted.

Patients with peripheral neuropathy of grade ≥1, brain metastases, concurrent therapeutic warfarin use, uncontrolled concurrent serious medical illnesses, pregnant or breast-feeding women, or patients with gastrointestinal malabsorption were not eligible to participate in the study.

Written informed consent was obtained from all patients, and the institutional review board of each participating hospital approved the study. This study was registered in the University Hospital Medical Network Clinical Trials Registry in Japan (UMIN000002797; http://www.umin.ac.jp/ctr/).

### Study design

This was an open-label, single-arm, multicenter, phase II study conducted at 24 academic centers in Japan. Treatment consisted of intravenous oxaliplatin (85 mg/m^2^) and l-leucovorin (l-LV; 200 mg/m^2^) administered intravenously over a 2-h period on day 1, followed by a bolus of 5-FU (400 mg/m^2^) and a 46-h infusion of 5-FU (2400 mg/m^2^), defined as the mFOLFOX6 regimen. Treatment cycles were repeated every 14 days.

Staging procedures were conducted every 8 weeks. Patients were removed from the study if they withdrew their consent, if they experienced unacceptable toxicity, if they had a treatment delay of >2 weeks because of toxicity from the treatment, or if the investigator deemed that withdrawal was in the patient’s best interest.

### Dose reductions

All toxicities were graded according to the National Cancer Institute Common Toxicity Criteria, version 3.0 (http://ctep.cancer.gov/protocolDevelopment/electronic_applications/ctc.htm), except for neurotoxicity. Initiation of a cycle of mFOLFOX6 required grade ≤1 granulocytopenia, grade ≤1 thrombocytopenia, and recovery from any treatment-related nonhematologic toxicity (excluding alopecia and neurosensory toxicity) to baseline or to grade ≤1.

Treatments with 5-FU and oxaliplatin were interrupted during a cycle if there was grade 3 or 4 hematologic toxicity (excluding anemia), or grade ≥2 nonhematologic toxicity (excluding nausea, vomiting, fatigue, or anorexia). The 5-FU dosage was reduced by 17% for grade 2 hand–foot syndrome, by 50% for grade 3 hand–foot syndrome, by 25% for grade 3 nonhematologic toxicity, by 50% for grade 4 nonhematologic toxicity, or by 25% for a delay in hematologic recovery of >1 week (excluding anemia). The 5-FU and oxaliplatin dosages were reduced, respectively, by 20 and 25% for grade 3 or 4 hematologic toxicity (excluding anemia), grade 3 or 4 nonhematologic toxicity (excluding nausea, vomiting, fatigue, or anorexia), or a delay in hematologic recovery of >1 week (excluding anemia). The oxaliplatin dosage was reduced by 25% for paresthesia with pain or functional impairment >7 days, and was discontinued if paresthesia with pain or functional impairment persisted throughout any treatment cycle.

### Statistical analysis

All analysis followed the intent-to-treat principle. The primary endpoint was 1-year PFS as assessed by the treating investigator. Responses were determined according to Response Evaluation Criteria In Solid Tumors (RECIST) (version 1.1) (http://www.irrecist.com). Secondary endpoints included overall response rate (ORR), OS, PFS, and safety. PFS and OS were defined as the time from the date of registration to the date of disease progression or death, respectively. PFS and OS were analyzed using the Kaplan–Meier method. The log-rank test was used to compare survival rates between groups. Multivariate analyses were performed using the Cox proportional hazards test. Toxicity data were analyzed in all patients who received at least one dose of study medication. Statistical analysis was performed by a statistician (KY) at an independent academic research organization. All statistical analysis was performed using SAS Release 9.13 (SAS Institute Inc., Cary, NC, USA). Initially, a total of 31 patients was determined to reject the 1-year PFS of 25% under the expectation of 45% with a power of 0.80 and a one-sided alpha of 0.10. However, on 23 October 2012, because of the slow accrual of patients, the sample size was amended to 24 patients for final analysis to provide a one-sided 90% confidence interval (CI) for the 1-year PFS rate, which would exclude a threshold value of 25% if the observed 1-year PFS was ~45%. Following the protocol amendment to sample size, the primary analysis was an evaluation of 1-year PFS using the Kaplan−Meier method relative to the pre-specified threshold values of 25%.

### Immunohistochemical analysis

Immunohistochemical (IHC) staining was performed on 5-μm-thick unstained sections from tissue microarray blocks using antibodies to cytokeratin 7 (CK7) (clone OV-TL12/30; Dako, Carpinteria, CA, USA; 1:300 dilution), to cytokeratin 20 (CK20) (clone Ks20.8; Dako; 1:200 dilution), to homeobox protein CDX2 (clone CDX2-88; Abcam, Cambridge, MA, USA; 1:100 dilution), and to epidermal growth factor receptor(EGFR) (clone 3C6; Roche Diagnostics, Mannheim, Germany; 1:100 dilution). IHC staining of human epidermal growth factor receptor 2 (HER2) was performed using the Ventana Ultra View DAB detection kit (Ventana Medical Systems, Tucson, AZ, USA) and the Ventana PATHWAY *HER2/neu* rabbit monoclonal antibody (4B5) on a Ventana BenchMark XT immunostainer (Ventana Medical Systems). All slides from each tumor were evaluated by a single pathologist independently based on the following criteria. Expression of CK7, CK20, and CDX2 was considered positive if 10% of the tumor cells showed immunoreactivity. For EGFR, both the percentage of positive tumor cells and the intensity of positive staining were graded according to a previous report [23]. Total grades were generated on a scale of 0–6 and considered positive if the score was 2–6. The staining for HER2 was graded according to the guidelines for such testing in gastric cancers [24].

## Results

### Baseline characteristics

Between April 2010 and November 2012, 24 patients with advanced SBA were enrolled from 12 institutions in Japan. The baseline characteristics of the intention-to-treat population are listed in Table 1. Metastatic disease was present in 22 of the patients, and the ECOG performance status was 0 or 1 in all patients. There were 14 patients with SBA in the duodenum (58%) and 10 patients with SBA in the jejunum (42%). The liver was the most common site of metastasis in 10 patients (40%). Three patients showed recurrence after curative resection of the SBA without receiving adjuvant therapy. Seven patients including the above recurrence cases underwent primary resection, and six underwent bypass therapy to resolve stenosis of the primary tumor. Eleven patients (46%) had not undergone any prior surgery. Four patients were excluded from the analysis of response rate (RR) because of lack of target lesion of RECST ver 1.1. The median follow-up time was 14.7 months (range 3.7–40.3).

**Table 1:** Clinical and pathological characteristics of the patients

	*N* = 24	(%)
Gender: male/female	18/6	75/25
Age: median, years (range)	63 (31–79)	
ECOG PS: 0/1/2	17/7/0	71/29/0
Disease status: locally advanced/metastatic	2/22	8/92
Metastatic site: liver/lung/peritoneum/distant lymph node/other	10/3/2/9/4	42/13/8/39/17
Primary tumor site: duodenum/jejunum/ileum	14/10/0	58/42/0
Histology: well to moderate/poor/muc/sig	17/4/2/1	71/17/8/4
CEA <5/5	15/9	63/38
CA19-9 <37/37	10/14	42/58
Prior surgery: primary resection/bypass/none	7/6/11	29/25/46
Prior adjuvant chemotherapy: none/yes	3/0	

*ECOG* Eastern Cooperative Oncology Group, *CA19-9* carbohydrate antigen 19-9, *CEA* carcinoembryonic antigen, *PS* performance status

### Chemotherapy and response

The ORR was 9/20 (45%) and the disease control rate was 16/20 (80%). One patient with liver metastasis had a complete response (CR) to mFOLFOX6. This patient started study treatment after undergoing resection of the SBA (pancreaticoduodenectomy), and we determined a CR after 20 cycles of chemotherapy. This patient was currently alive without evidence of disease at 12 months after the initiation of treatment. Second-line chemotherapy was received by 12 patients, while six patients received additional mFOLFOX6 therapy.

### Efficacy

**Fig. 1:**
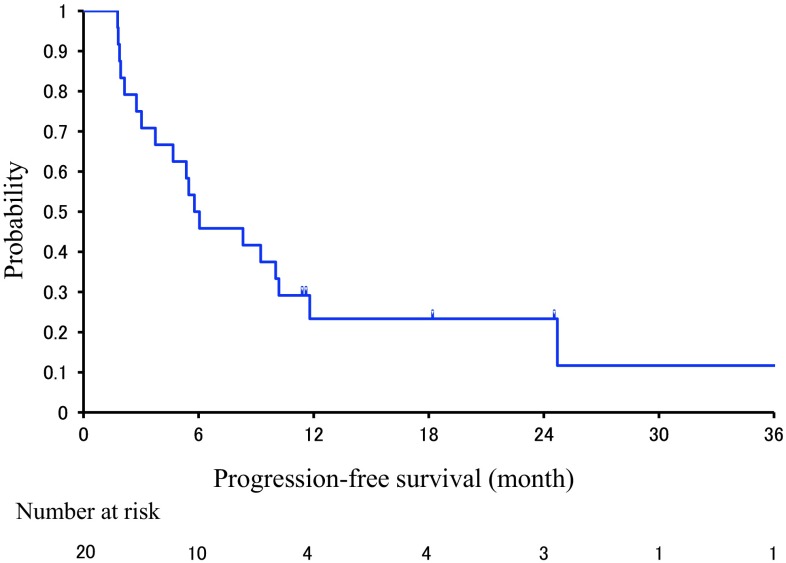
The survival rate in this study: progression-free survival (PFS) curve

**Fig. 2:**
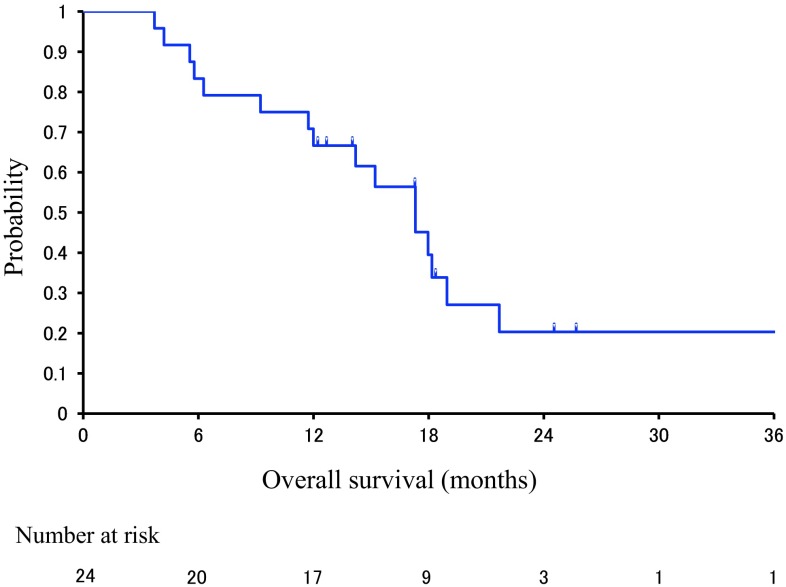
The survival rate in this study: overall survival (OS) curve

The primary endpoint for this study was 1-year PFS as assessed by the treating investigator. With a median follow-up of 14.7 months, the PFS rate at 1 year was 23% (95% CI 8.6–44.2%). The median PFS and OS were 5.4 months (95% CI 4.8–6.0 Fig. 1), and 17.3 months (95% CI 11.7–19.0 Fig. 2), respectively. An exploratory analysis was conducted to determine the prognosis factors that might have influenced OS (Table 2). Parameters studied included age, PS, primary site and histological grade of the tumor, resection of the primary tumor or bypass, numbers of metastatic organs, and serum carcinoembryonic antigen (CEA) and carbohydrate antigen (CA)19-9 levels. Upon univariate analysis, good PS (0), location in the jejunum, resection of the primary tumor or bypass, and a low level of serum CEA (<5 ng/mL) were all significantly associated with longer OS. However, upon multivariate analysis, only resection of primary tumor or bypass was an independent predictor of better OS (*P* = 0.023). The RR was slightly higher in patients with a tumor in the jejunum (4/7; 57%) than in the duodenum (5/13; 38%).

**Table 2:** Univariate and multivariate analysis of factors associated with survival

	Univariate			Multivariate	
	*N*	HR (95% CI)	*P*	HR (95% CI)	*P*
ECOG PS					
0	17				
1	7	3.69 (1.31–10.38)	0.014	2.60 (0.73–9.28)	0.14
Primary site					
Jejunum	10				
Duodenum	14	2.88 (0.89–9.27)	0.077	2.85 (0.85–9.52)	0.090
Prior surgery					
Yes	13				
No	11	2.83 (1.02–7.84)	0.046	3.98 (1.21–13.07)	0.023
Serum CEA (ng/mL)					
<5	15				
≥5	9	2.51 (0.90–7.01)	0.079	1.61 (0.43–6.00)	0.48

*CEA* carcinoembryonic antigen, *CI* confidence interval, *ECOG* Eastern Cooperative Oncology Group, *HR* hazard ratio, *PS* performance status

An exploratory analysis was conducted to determine the characteristic factors of the patients that might have influenced response. However, no difference between the responder and non-responder patients was identified for age, gender, PS, histological grade, tumor resection or bypass, location of primary tumor and metastasis, serum CEA and CA19-9 levels.

### Toxicity

All 24 patients who received one dose of the study treatment were evaluated for toxicity. The common treatment-related grade 3–4 adverse events are listed in Table 3. The most common events were neutropenia (38%), anemia/peripheral neuropathy (25%), stenosis (17%), fatigue/anorexia/bilirubin increase (8%), and diarrhea (4%). One nonhematological grade 4 toxicity occurred; this patient had symptomatic cerebrovascular ischemia and was under treatment for type 2 diabetes and hypertension, and discontinued chemotherapy. There were no treatment-related deaths.

**Table 3:** The most common treatment-related toxicities based on the Common Terminology Criteria for Adverse Events (CTCAE; Ver. 3.0)

	Any grade *N* (%)	Grade 3/4 *N* (%)
Neutropenia	12 (50)	9 (38)
Anemia	13 (54)	6 (25)
Thrombocytopenia	10 (42)	0 (0)
Fatigue	18 (75)	2 (8)
Nausea	18 (75)	0 (0)
Vomiting	9 (38)	0 (0)
Diarrhea	11 (46)	1 (4)
Stomatitis	5 (21)	0 (0)
Bilirubin elevation	4 (17)	2 (8)
Peripheral neuropathy	19 (79)	6 (25)
Hand–foot syndrome	4 (17)	0 (0)
Pneumonitis	2 (8)	0 (0)
Stenosis	9 (38)	4 (17)
Hemorrhage	1 (4)	1 (4)
Cerebrovascular ischemia	1 (4)	1 (4)

### Immunohistochemistry

We were able to collect 13 tumor samples from the primary site and performed IHC analysis.

The most commonly expressed immunophenotypic marker was CDX2, observed in seven patients (54%). Expression of CK20 occurred in 5 (38%) patients, and expression of CK7 occurred in 4 (31%) patients. The tissues demonstrated great variability for CK7, CK20, and CDX2. There was no significant difference in the expression of CK7, CK20, and CDX2 between the duodenal and nonduodenal SBA. Expression of EGFR score 2–6 was observed in 3 (23%) patients. Expression of HER2 was not observed in this analysis.

There was no significant association between immunophenotypes and patient survival.

## Discussion

In the treatment of patients with advanced SBA, no prospective studies evaluated the benefit of chemotherapy compared with best supportive care. Single-institution retrospective studies have suggested that palliative chemotherapy confers a survival benefit to such patients [6, 8, 9]. In the largest retrospective analysis that evaluated 113 patients with advanced SBA, palliative chemotherapy predicted OS in a multivariate analysis (hazard ratio [HR] 0.47) [8]. Therefore, although no prospective study has evaluated outcomes, palliative chemotherapy is considered a standard treatment for patients with unresectable or recurrent SBA. In the past decades, these patients had been treated with the same chemotherapy regimen used for colorectal cancer (CRC) or gastric cancer. Therefore, in retrospective studies on patients with SBA, the most common regimen was 5-FU or 5-FU with a platinum agent (Table 4). Among them, combination chemotherapy of 5-FU with platinum compounds including oxaliplatin seemed to be more effective than other regimens.

**Table 4:** Summary of previous studies on patients with SBA

Authors	Study Type	Pts No.	Regimen	RR (%)	PFS/TTP (M)	MST (M)
Xiang et al. [22]	P II	33	FOLFOX	48.5	7.8	15.2
Tsushima et al. [20]	Retro	22	FOLFOX	42	9.6	22.2
Zaanan et al. [19]	Retro	38	FOLFOX	34	6.9	17.8
Overman et al. [21]	P II	30	CAPOX	50	11.3	20.4
Suenaga et al. [15]	Retro	10	5-FU-based	10	2.9	12
Overman et al. [18]	Retro	29	5-FU and Platinum	46	8.7	14.8
Aparicio et al. [32]	Retro	21	FOLFOX	NR	7	NR
Czaykowski et al. [9]	Retro	37	5-FU-based	5	NR	15.6
Fishman et al. [8]	Retro	44	Various	29	NR	18.6
Gibson et al. [11]	P II	39	FAM	18	5.0	8
Locher et al. [13]	Retro	20	5-FU and Platinum	21	8.0	14
Dabaja et al. [6]	Retro	48	NR	NR	NR	11
Crawley et al. [14]	Retro	8	ECF or 5-FU	38	7.8	13
Jigyasu et al. [16]	Retro	14	5-FU-based	7	NR	9
Morgan and Busuttil [33]	Retro	7	5-FU-based	0	NR	NR
Rochlin et al. [34]	Retro	11	5-FU	36	3.8	NR

*5-FU* fluorouracil, *ECF* epirubicin, cisplatin and 5-FU, *FAM* 5-FU adriamycin and mitomycin, *FOLFOX* oxaliplatin, leucovorin and 5-FU, *CAPOX* capecitabine and oxaliplatin, *RR* response rate, *PFS* progression-free survival, *TTP* time to progression, *MST* median survival time

Our study demonstrated an RR of 45%, a median PFS of 5.4 months, and a median OS of 17.3 months in patients given the mFOLFOX6 regimen. To our knowledge, only two prospective phase II studies have used the combination of fluoropyrimidine and oxaliplatin. Overman et al. reported on a capecitabine and oxaliplatin (CAPOX) regimen [21] and Xiang et al. reported on a mFOLFOX6 regimen but omitted a bolus 5-FU treatment [22]. The RR and median OS were similar in these studies. However, the PFS of our study was worse than that of the CAPOX regimen. Ono possibility is that patients with duodenal cancer were more frequently seen in our study than in the CAPOX study (58 vs 23%). Although there was no statistical difference in multivariate analysis for OS, the RR for patients with a tumor in the duodenum appeared lower than in the jejunum (38 vs 57%, respectively), and location in the jejunum was significantly associated with a longer OS in univariate analysis (*P* = 0.077). The primary site was demonstrated as a predictive or as a worse factor for OS in previous analyses and our study, respectively [25, 26]. This difference might reflect the heterogeneous nature of the epithelium of tumor origin between the duodenum and jejunum.

This study did not meet the primary endpoint because of the small sample size. However, the differences in PFS between the two prospective studies (above) and our result are small. Therefore, the survival benefit of combining fluoropyrimidine and oxaliplatin combination was confirmed as a first-line chemotherapy regimen for patients with unresectable or recurrent SBA.

In multivariate analysis, only resection of the primary tumor or bypass was an independent predictor of better OS (*P* = 0.023). Severe stenosis of grade ≥3 which requests resection of the primary tumor or bypass occurred in 4 patients (17%, 4/23) in this study. Resection of the primary tumor was also demonstrated to be a factor predictive of a better OS in other reports [18]. Palliative tumor resection has been traditionally advocated in metastatic CRC to prevent symptoms and complications linked to the primary tumor, such as obstruction, perforation, or bleeding. The risk of obstruction caused by the tumor during initial chemotherapy was 6–29% in patients with CRC [27, 28]. In this study, severe stenosis (grade ≥3) occurred in 3 patients (27%, 3/11) among the patients without resection of the primary tumor or bypass. All cases occurred within 2 months after starting chemotherapy. If the lumen of the small intestine is narrower than the colorectum, then we should consider that stenosis is likely to occur at an early stage of chemotherapy.

There were some limitations to our study. First, the small sample size hampered comparisons between subgroups. Nevertheless, to separate the heterogeneity in the survival outcomes associated with the primary site (duodenum vs jejunum) and prior surgery (resection of the primary tumor or bypass vs without resection), an exploratory analysis of the subgroups were performed. Second, IHC analysis used only a small subsample of the patients. No information was obtained from this analysis. Consequently, firm conclusions cannot be drawn from the subgroup and IHC analyses because the rarity of this disease hampers large-scale studies.

Importantly, this trial demonstrates the feasibility of the completion of phase II studies in such rare tumor types, and should promote more robust research on these orphan tumors. In addition, given the overall tolerability of the regimen, it is logical to investigate the role of targeted therapies in combination with mFOLFOX6.

The main challenge for the future will be to identify a molecular marker involved in small-bowel carcinogenesis that can predict chemosensitivity, and thus improve patient survival. In patients with unresectable CRC, the addition of bevacizumab to mFOLFOX6 was found to prolong PFS compared with mFOLFOX6 alone, and clinical trials that investigate mFOLFOX6 in combination with agents targeting angiogenesis would be reasonable for patients with SBA [29]. In addition, activation of mutations in the *KRAS* (or *RAS*) oncogene occur at a similar frequency in both SBA and colorectal adenoma tumors, which suggests a potential role for EGFR inhibition in a subset of patients with SBA [30, 31].

The infrequency of SBA made it difficult to conduct a prospective study. It is extremely unlikely that a randomized trial comparing two chemotherapy regimens could be undertaken. However, we have conducted this prospective phase II trial within a new cooperative group in Japan. It provides important insights for the treatment of patients with SBA. The mFOLFOX6 regimen proved effective and probably represents a new standard treatment for patients with an unresectable or recurrent SBA. In the future, the mFOLFOX6 regimen in combination with molecular targeting therapy should be evaluated prospectively to improve the outcomes even for patients with SBA; however, worldwide multi-institutional cooperation will be necessary for investigating such a rare disease.
